# X-ray phase contrast tomography for the investigation of amyotrophic lateral sclerosis

**DOI:** 10.1107/S1600577520006785

**Published:** 2020-06-09

**Authors:** Ginevra Begani Provinciali, Nicola Pieroni, Inna Bukreeva, Michela Fratini, Lorenzo Massimi, Laura Maugeri, Francesca Palermo, Fabrizio Bardelli, Alberto Mittone, Alberto Bravin, Giuseppe Gigli, Francesco Gentile, Andrea Fossaghi, Nilo Riva, Angelo Quattrini, Alessia Cedola

**Affiliations:** a Physics Department ‘Sapienza’ University, CNR-Institute of Nanotechnology, Piazzale Aldo Moro 5, 00185 Rome, Italy; b Laboratoire d’Optique Appliquée, ENSTA Paris Tech, 828 Boulevard des Maréchaux, 91120 Palaiseau, France; c Fondazione Santa Lucia IRCCS, Via Ardeatina 306, 00179 Rome, Italy; d Department of Medical Physics and Biomedical Engineering, University College London, Gower Street, London WC1E 6BT, United Kingdom; e Dipartimento di Fisica, Università della Calabria, Via P. Bucci, Cubo 31 C, 87036 Arcavacata di Rende (Cosenza), Italy; f European Synchrotron Radiation Facility, 71 Avenue des Martyrs, 38043 Grenoble, France; g CNR Nanotec, Institute of Nanotechnology, via Monteroni, 73100 Lecce, Italy; h Dipartimento di Matematica e Fisica, Universita’ del Salento, via Arnesano, 73100 Lecce, Italy; i Neuropathology Unit, Institute of Experimental Neurology, Division of Neuroscience, San Raffaele Scientific Institute, 20132 Milan, Italy

**Keywords:** X-ray phase contrast tomography, ALS, spinal cord

## Abstract

Amyotrophic lateral sclerosis (ALS) is a progressive neurodegenerative disorder affecting motor neurons. Pre-clinical studies drive the development of animal models that well mimic ALS disorder and enable both the dissection of disease processes and an early assessment of therapy efficacy. A comprehensive knowledge of neuronal and vascular lesions in the brain and spinal cord is an essential factor to understand the development of the disease. Spatial resolution and bidimensional imaging are important drawbacks limiting current neuroimaging tools, while neuropathology relies on protocols that may alter tissue chemistry and structure. In contrast, recent ex vivo studies in mice demonstrated that X-ray phase-contrast tomography enables study of the 3D distribution of both vasculature and neuronal networks, without sample sectioning or use of staining. Here we present our findings on ex vivo SOD1^G93A^ ALS mice spinal cord at a micrometric scale. An unprecedented direct quantification of neuro-vascular alterations at different stages of the disease is shown.

## Abstract 2

Ex vivo X-ray phase contrast tomography of amyotrophic lateral sclerosis of a SOD1^G93A^ mouse model is presented. Quantification of neuronal and vascular alteration in the central nervous system is described.

## 1. Introduction   

ALS is the most common and severe form of motor neuron diseases (MND), a heterogeneous group of neurodegenerative disorders involving motor neurons (MNs). Progressive paralysis and respiratory failure rapidly lead to death, usually occurring two to five years after diagnosis (Riva *et al.*, 2016 ▸). Riluzole and edavarone are the only approved drugs for therapy, but their beneficial impact on clinical management is low or doubtful (Hardiman *et al.*, 2017 ▸; Es *et al.*, 2017 ▸).

Hallmark neuropathologic features in ALS are the loss of upper and lower motor neurons (UMN/LMNs) and their fibers in the primary motor cortex, brainstem and anterior horns of the spinal cord (Saberi *et al.*, 2015 ▸), together with the presence of phospho­rylated TAR DNA-binding protein 43 (TDP-43), positive cytoplasmic inclusions in both MNs and glial cells (Brettschneider *et al.*, 2013 ▸).

Despite most ALS cases apparently occurring sporadically (sALS), up to 10% show familial inheritance (fALS) (Kiernan *et al.*, 2011 ▸). Several disease mechanisms have been proposed to describe ALS pathogenesis, such as dysregulation of RNA processing or pathological protein aggregation (Riva *et al.*, 2016 ▸; Bonafede & Mariotti, 2017 ▸). Nevertheless, the etiology and pathogenesis of ALS remain unknown. Superoxide dismutase 1 (SOD1) was the first identified ALS gene (Rosen *et al.*, 1993 ▸), upon which mouse models resembling ALS have been generated and extensively studied.

Both neuronal and non-neuronal mechanisms concurrently participate in ALS pathogenesis (Hardiman *et al.*, 2017 ▸). Among the latter case, evidence of blood brain/spinal cord barrier (BBB/BSCB) breakdown has recently emerged, showing an increased vascular permeability (Miyazaki *et al.*, 2011 ▸) and reduced vascularization along the disease course (Kew *et al.*, 1993 ▸; Rule *et al.*, 2010 ▸). Nonetheless, their relationship with MN degeneration has not been fully elucidated.

In recent years, the evolution of imaging technologies allowed for in vivo visualization of the nervous system in affected ALS patients and animal models (Chiò *et al.*, 2014 ▸; Marcuzzo *et al.*, 2017 ▸). Structural and functional assessment through different neuroimaging tools, including magnetic resonance imaging (MRI) and its variants, allowed replication of most of the phenomena observed in ALS, such as atrophy of motor cortex and anterior horns (Cohen-Adad *et al.*, 2013 ▸) increasing reactive gliosis (Turner *et al.*, 2004 ▸), spreading of pathology along functionally connected areas (Schmidt *et al.*, 2016 ▸), and neurovascular alterations (Evans *et al.*, 2014 ▸). However, spatial resolution and bidimensional sectioning represent important limits, affecting its sensibility in early detection of the disease, when damage is still restricted to micro-environmental spots in the cortex or spinal cord (Bodansky *et al.*, 2010 ▸).

In contrast, recent works demonstrated that X-ray phase-contrast tomography (XPCT) enables the visualization of the 3D distribution of neuronal and vascular network of the whole spinal cord (Fratini *et al.*, 2015 ▸; Cedola *et al.*, 2017 ▸), with a micrometric to sub-micrometric spatial resolution with a simple and rapid sample preparation. In conventional radiography, the collected transmitted intensity is formed by the interaction of the incident X-ray beam with absorbing structures inside the sample. Phase contrast imaging is an alternative technique that exploits phase variations, produced by spatial variations of the real part of the refractive index, induced by the sample on the incident coherent/partial coherent X-ray beam.

Herein, we applied XPCT 3D imaging to the SOD1^G93A^ ALS mouse, providing a 3D morphological description of the progressive loss of neuronal and vascular network in the spinal cord.

## 2. Materials and methods   

### 2.1. Animal model and samples preparation   

Experiments were performed on transgenic mutant SOD1 mice carrying the SOD1^G93A^ allele (strain B6SJL-TgN [SOD1-G93A]1GUR) (Jackson laboratories) and wild-type (WT) non-transgenic littermates. Based on each animal’s age and behavioral studies, SOD1^G93A^ mice were divided into two groups according to disease stage and corresponding to the following time points: 60 days, presymptomatic stage; 90 days symptomatic stage (initiation of body weight loss) (*n* = 3 mice per time point). Sixty and 90 days-old WT animals were used as age-matched controls. Mice were transcardially perfused with heparin and physiological solution. Afterwards, spinal cord was dissected out, fixed in 4% paraformaldehyde for 24 h, and then maintained in 70% alcohol until analysis. To investigate L2 and L3 spinal segments with major motor neuron clusters, mice underwent lamine­ctomy centered at the tenth thoracic vertebra and the spinal cord was taken off, dehydrated using graded ethanol and immersed in methyl salicylate for 24–48 h.

Animal experimentation was approved by the Italian Ministry of Health and carried out in agreement with the institutional guidelines and international laws (Directive 2010/63/EU on the protection of animals used for scientific purposes, transposed into the Italian legislation by the ‘Decreto Legislativo’ of 4 March 2014, n. 26).

### 2.2. X-ray phase-contrast tomography   

#### 2.2.1. Experimental set-up   

All the samples were measured at beamline ID17 of ESRF in Grenoble, France. The experiment on ALS-affected mice spinal cords was performed with pink beam (spectrum peaked at 44 keV); 2000 projections covering a total angle range of 360° were acquired by using an acquisition time of 1 s per point. Spinal cord samples were set at 2.3 m from a PCO.Edge5.5 sCMOS detector coupled with optics systems to obtain a final pixel size of 3.06 µm. The corresponding field of view was 7.6 mm × 6.4 mm.

In-line free space propagation imaging is based on Fresnel’s diffraction patterns after free space propagation (Di Fonzo *et al.*, 1998 ▸; Fratini *et al.*, 2015 ▸). Despite the advantage offered by the easier experimental set-up compared with complex optical systems, propagation images contain both absorption and phase contribution. In order to disentangle the two effects a specific algorithm has been used to decouple absorption from phase information. A single-distance phase-retrieval algorithm (assuming an homogeneous sample and δ/β = constant) proposed by Paganin *et al.* (Paganin, 2006 ▸) was applied to all acquired tomographic projections.

Data pre-processing, phase retrieval and reconstruction were performed using the *SYRMEP Tomo Project* software (Cedola *et al.*, 2017 ▸; Massimi *et al.*, 2018 ▸). The different electron densities of the tissues were rendered as gray levels in the phase tomograms images. To independently display the different tissues, image analysis and image segmentation have been performed using both free software (*ImageJ*), high-end software (*VGSTUDIO MAX*) and custom-made scripts implemented in *ImageJ*.

#### 2.2.2. Quantitative analysis of XPCT images   

We focus our analysis on the lumbar region of the spinal cord (0.3 mm × 0.3 mm × 1.3 mm). We decided to analyze the lumbar-sacral region of the spinal cord because the Rexed lamina IX of the ventral horn is larger here. It is possible to differentiate several bright structures in the XPCT images (see Fig. 2): meninges and spinal roots that anatomically surround the spinal cord, as well as neuronal cells and veins.

**Figure 1:**
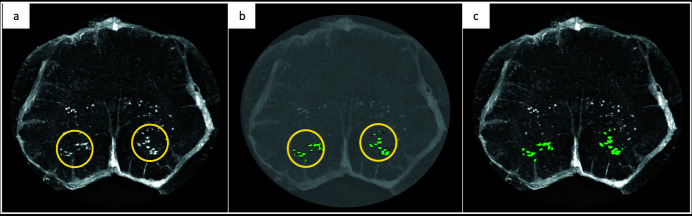
X-ray phase contrast tomography images of the lumbar region of the spinal cord. Example of the MN 3D segmentation process. (*a*) A ROI was selected in the ventral horn of the spinal cord where the MNs are anatomically concentrated. (*b*) An intensity threshold segmentation was performed to isolate MNs. (*c*) Superimposed image of (*a*) and (*b*) to show the output of the segmentation process.

MNs’ quantification was performed through an automated counting process, using the 3DObjectsCounter (Bolte & Cordelières, 2006 ▸; https://imagej.net/3D_Objects_Counter) *ImageJ* plug-in (Fig. 1 ▸). We selected a region of interest (ROI) in the ventral horn of the spinal cord where the MNs are anatomically concentrated and their size and position were compared with the literature and histological images. Neuron quantification was carried out in several steps: (1) an intensity threshold was performed, since neurons appear as bright objects in XPCT images; (2) on the binarized images we applied the 3D object counter plug-in – this plug-in takes as input the volume of the object of interest (in pixels) and counts the number of 3D objects in a stack.

The vascular network was visualized thanks to the detection of the veins and capillaries inside a selected volume of the lumbar region of the spinal cord.

The segmentation of the vascular network was performed through a 3D image segmentation process. Starting from the 3D tomographic reconstruction image, we apply an intensity threshold segmentation to isolate the vascular network (appearing as white tubular objects, as seen in the inset at 60 days in Fig. 4). On these binarized images, we then apply the Skeletonize *ImageJ* plug-in (Arganda-Carreras *et al.*, 2010 ▸; https://imagej.net/AnalyzeSkeleton) to calculate the number of branches per mm^3^. This plugin tags all pixel/voxels in a skeleton image. The voxels are classified into three different categories depending on their 26 neighbors: end-point voxels – if they have less than two neighbors; junction voxels – if they have more than two neighbors; slab voxels – if they have exactly two neighbors. The number of branches is the number of slab segments, usually connecting end-points, end-points and junctions or junctions and junctions.

The percentage of MN loss and vascular density in diseased animals was compared with control samples in the pre-symptomatic and symptomatic stages.

### 2.3. Neuron lesions   

**Figure 2:**
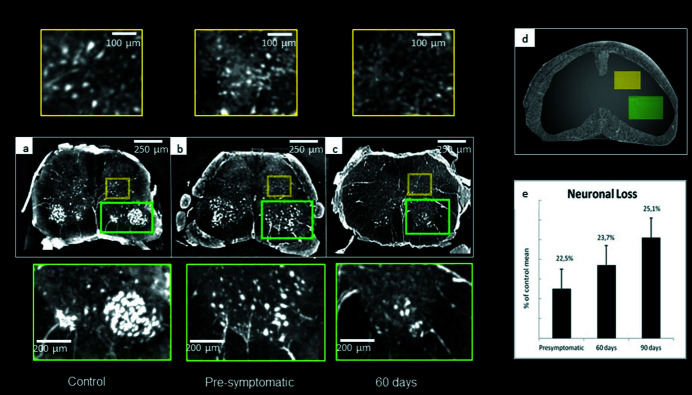
X-ray phase contrast tomography images of the lumbar region of the spinal cord. Reconstructed volumes (about 1 mm thick) of the lumbar region of the spinal cord. Axial sections of healthy (*a*), pre-symptomatic (*b*) and 90 days ALS affected (*c*) samples. Cell loss induced by ALS progression can be observed in (*b*) and *(c*). Lower inset (green): magnification of ROIs in the ventral horn of the spinal cord containing neuronal cells compatible with motor neurons. Upper insets (yellow): magnification of ROIs to show neuronal cells compatible with sensor interneurons. In a pre-symptomatic stage, the number of the sensory neurons remains almost unaltered compared with the control sample, while a decrease in cell number is shown in the 90 days SOD1 mice. Images are obtained with a pixel size of 3.06 µm at ID17 at the ESRF. (*d*) Schematic representation of the selected ROIs shown in (*a*, *b*, *c*): quantitative analysis was performed in the ventral horn (green) of the spinal cord. (*e*) Percentage of the number of neurons in ALS samples compared with the control samples in the ventral horn at different time-points. Error bars represent standard deviation of data.

Neuronal lesions were evaluated at different time-points of the disease through XPCT experiments on mice lumbar spinal cords affected by ALS and were compared with the control samples. Fig. 2 ▸ summarizes the results. MNs appear as very bright objects inside the tomographic volume and it is therefore possible to segment them by applying an intensity threshold. An axial view of the reconstructed tomographic images for the control [Fig. 2(*a*) ▸], pre-symptomatic SOD1^G93A^ 60 days old Fig. 2(*b*) ▸] and 90 days old SOD1^G93A^ [Fig. 2(*c*) ▸] mice spinal cords are shown. Images were acquired with a spatial resolution of about 9 µm. Although the results were confirmed in all the samples for each category, only one image per category has been reported. The insets at the top of Fig. 2 ▸ represent a magnification of the neuronal cells obtained through maximum intensity projection method. In order to better visualize the region of interest for our analysis, we provide a sketch [Fig. 2(*d*) ▸] of the spinal cord with the two analyzed regions depicted in yellow (where the interneurons are present) and in green (where the MNs are located). As expected, the number of MNs in the ventral horns decreased compared with the control at the time of symptom onset (90 days), as shown in Fig. 2 ▸ and summarized in the bar chart [Fig. 2(*e*) ▸]. Furthermore, at a pre-symptomatic stage (60 days) the loss of motor neurons appears already evident, as appreciated in the bottom inset of Fig. 2(*b*) ▸, compared with the control case.

**Figure 3:**
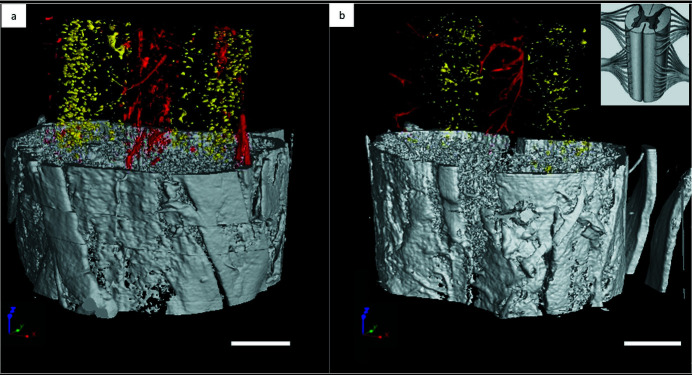
3D reconstruction of the vascular network (segmented in red) and the neuronal network (segmented in yellow) of healthy (*a*) and ALS-affected (*b*) mice spinal cord (L2-L3 segment). Samples were approximately 60 days old. Inset: schematic representation of the anatomical orientation of the spinal cord. Scale bar: 400 µm. The pixel size is 3.06 µm.

In the pre-symptomatic stage, the number of neurons in the posterior horns (probably sensory interneurons) of SOD1^G93A^ mice is almost unaltered compared with WT mice, although their size appears reduced, as shown in the top inset of Fig. 2(*b*) ▸. Following, at 90 days old a reduction in neurons can be appreciated in the ROI of SOD1^G93A^ compared with WT mice, while small cells compatible with inflammatory cells occupy this region, as reported in the top inset of Fig. 2(*c*) ▸. In Fig. 3 ▸, 3D rendering images of 60 days old WT and SOD1^G93A^ mice are shown, offering a more visual proof of the reduction in the distribution and density of neurons (yellow) and blood vessels (red) in the pre-symptomatic stage of SOD1^G93A^ mice compared with controls.

### 2.4. Vascular lesions   

**Figure 4:**
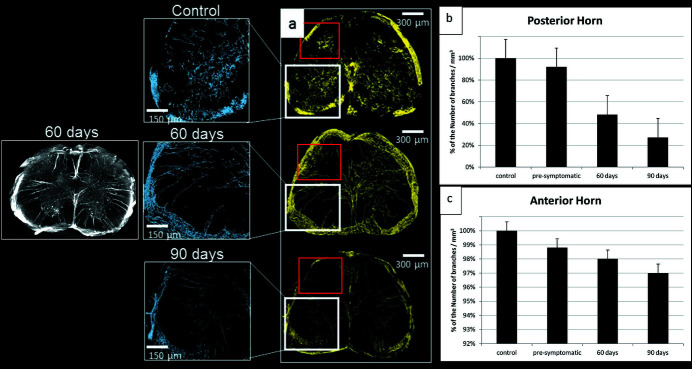
Vascular segmentation (about 1 mm thick) of the lumbar region of the spinal cord. (*a*) Vascular network segmentation obtained by Skeletonize *ImageJ* plug-in. White inset: magnification of the selected ROI in the ventral horn of the spinal cord. Red inset: analyzed area in the dorsal horn. A maximum intensity projection image is shown for the 60 days case. Quantification of vascular distribution as the percentage of the number of branches per mm^3^ in the posterior horn (data normalized to the control sample) (*b*) and in the Anterior horn (data normalized to the control sample) (*c*).

We performed a segmentation of the same XPCT images used in Section 2.3 in order to isolate the vasculature (Fig. 4 ▸, rendered in yellow, blue in the insets) in the ventral and dorsal horn of WT [Fig. 4 ▸(*a*), top] and 60 and 90 days old SOD1^G93A^ mice [Fig. 4(*a*) ▸, middle and bottom, respectively]. The image of the pre-symptomatic sample is not shown because no apparent vascular alteration was found. However, in the latter case, a slight alteration compared with the control case was found as a result of the quantitative analysis of vascularization in the ventral horn [Fig. 4(*b*) ▸]. Three mice for each category have been measured and the most significant one is reported here. The images reported in Fig. 4 ▸ display the segmented vessels in the XPCT axial reconstructions: insets on the left show a magnification of the ventral horn. We implemented a gray-level segmentation process in order to perform a quantitative analysis that allowed us to make a comparison between the different time-points and the control. The number of branches per mm^3^ was calculated, data were normalized with respect to the control sample and expressed as percentage values. A few percentage points of decreasing in the vasculature, compared with the control, are evident as the time course of the disease progresses [Fig. 4(*b*) ▸]. On the other hand, a considerable loss in the vessel branches was found in the posterior horn of SOD1^G93A^ mice compared with WT samples at the same time points, with a progressive reduction observed also between pre-symptomatic and symptomatic mice [Fig. 4(*c*) ▸].

## 3. Discussion   

An advancement in imaging technology is necessary to achieve important breakthroughs in the comprehension of disease pathogenesis and clinical management in several fields of medicine. In particular, neurodegenerative disorders necessitate in-depth investigation of disease mechanisms at both the macro- and microscopic level. This, in turn, requires tools enabling a direct and comprehensive visualization of disease-affected neurons and their vascularity.

In the present study, we exploit XPCT 3D images to investigate the alterations in vascular and neuronal networks in the lumbar region of the spinal cord, providing a direct 3D morphological description of CNS lesions in the most studied ALS animal model, the SOD1^G93A^ mouse, using a spatial resolution of about 9 µm (isotropic voxel size of 3.06 µm).

Highly coherent synchrotron X-ray sources can be used for non-conventional contrast mechanisms. Phase contrast imaging exploits phase variations produced by spatial variations of the real part of the refractive index induced by the sample on the incident coherent/partial coherent X-ray wave. Different imaging techniques have been developed to detect phase contrast (Diemoz *et al.*, 2012 ▸). This technique is particularly useful when imaging weakly absorbent materials as it enables greater contrast sensitivity in the discrimination of details with similar densities, increasing the signal-to-noise ratio up to 1000 times compared with absorption-contrast CT images (Momose *et al.*, 1996 ▸).

Furthermore, XPCT requires standard ex vivo sample preparation. In fact, while fixation is needed for tissue preservation, further processing (like paraffin/resin embedding and staining) is not required.

The application of XPCT technology proved to be highly reliable in the evaluation of normal and pathologic nervous tissues, including several neurodegenerative disease animal models (Bukreeva *et al.*, 2017 ▸; Cedola *et al.*, 2017 ▸; Massimi *et al.*, 2018 ▸). Here, we showed that this technique highly replicates the loss of MNs in the ventral horns of the SOD1^G93A^ spinal cord. Previous studies using classic pathology demonstrated that MN loss starts at the time of symptom onset (90 days), with a 40% reduction in the number of neurons from 80 to 100 days while no significant change occurs before the appearance of clinical manifestations (Dai *et al.*, 2017 ▸). Through XPCT, a similar percentage in neuronal loss can be shown at the same time point, confirming the evidence present in the literature. However, we showed that MN loss does not start at the time of symptom onset but is rather present already in the pre-symptomatic stage (60 days), although to a less extent. Such discordance with previous studies may root from the different methodologies: classic histology shows several limitations and drawbacks in the comprehensive evaluation of neuronal loss in the spinal cord, as multiple sections and 2D representation may lead to missing information. On the other hand, XPCT allows a reliable analysis of the spinal cord, avoiding the loss of important data and identifying signs of disease when it is still confined to local spots in the CNS. Furthermore, the choice of ALS animal model may influence the findings. Although in the classic SOD1^G93A^ model the MN loss appears limited, in mice models with more prolonged disease a MN number reduction is observed already in the pre-symptomatic stage (Feeney *et al.*, 2001 ▸; Acevedo-Arozena *et al.*, 2011 ▸), supporting the hypothesis that the discrepancy between our results and the previous literature is due to methodological issues rather than different findings.

Disruption of the BB/BSC vascular barrier has recently emerged as a novel contributor in ALS pathogenesis. Loss of vascular density and increase in vessel permeability have been demonstrated in both animal and human ALS (Garbuzova-Davis *et al.*, 2011 ▸), with signs of pathology being present in the early stage, even prior to MN loss, and progressively worsening thereafter (Garbuzova-Davis *et al.*, 2007 ▸). Herein, we showed that the vascular network is significantly reduced in both the ventral and dorsal horns of the SOD1^G93A^ spinal cord mice. The breakdown of BSCB may have important implications in ALS, as the undernourishment caused by the decrease in vascular distribution may trigger or aggravate MN degeneration.

## 4. Conclusions   

The XPCT 3D technique was found to be a unique tool in the investigation of neurodegenerative diseases. Here we attest that XPCT can provide an unprecedented direct quantification of neurovascular alterations in SOD1^G93A^ ALS mice at different stages of the disease. We demonstrated the ability to detect neuron alterations even in a pre-symptomatic state. X-ray phase contrast tomography of biological tissues significantly complements conventional 2D methods capability, such as classical histology, or more modern developments such as fluorescence light-sheet microscopy. It allows a direct multiscale approach for full 3D digitalization of scalable tissue volumes and provides a novel contrast, which enables both the imaging of low and high-density tissues highlighting the small density variations inside the sample. All these traits make XPCT a novel essential tool in neurodegenerative research.
